# Psychometric evaluation of the Chinese version of the Positive Health Behaviours Scale for clinical nurses: a cross-sectional translation

**DOI:** 10.1186/s12912-023-01453-z

**Published:** 2023-08-31

**Authors:** Linghui Kong, Tingting Lu, Chen Zheng, Huijun Zhang

**Affiliations:** Departement of Nursing, Jinzhou Medical University, Jinzhou, China

**Keywords:** Clinical nurses, Factor analysis, Health behaviours, Occupational health, Psychometric evaluation

## Abstract

**Background:**

Occupational health is essential for nurses in clinical nursing practice. However, there is no specific tool for measuring the health behaviour of clinical nurses in China. This study aimed to translate the Positive Health Behaviours Scale into Chinese and validate its psychometric properties among clinical nurses.

**Design:**

A cross-sectional design with repeated measures.

**Methods:**

A total of 633 clinical nurses were recruited by convenience sampling from hospitals in Liaoning Province, China. After obtaining the authorization of the original author, the PHBS was translated into Chinese by the Brislin back-translation method. Item analysis was completed to evaluate item discrimination, and the Delphi method was adopted to analyse content validity. Exploratory factor analysis and confirmatory factor analysis were conducted to explore and validate the underlying factor structure. Internal consistency and test-retest reliability were calculated to evaluate reliability.

**Results:**

A total of 29 items were retained in the item analysis, and the content validity index of the translated scale was 0.956. In the EFA, four common factors were extracted (nutrition, physical activity, relaxation and behaviours related to mental health and preventive behaviours), explaining 60.81% of the total variance. The results of the CFA were as follows: χ^2^/df = 1.363, GFI = 0.902, NFI = 0.909, IFI = 0.974, TLI = 0.971, CFI = 0.974, RMSEA = 0.034, and RMR = 0.023. The results of the EFA and CFA showed that the translated scale had good structural validity. Cronbach’s α coefficient, the split-half reliability and the test-retest reliability of the Chinese version of the PHBS were 0.928, 0.953 and 0.891, respectively. At the same time, the translated scale had good reliability.

**Conclusions:**

The Chinese version of the PHBS for clinical nurses had good psychometric properties. The results of the questionnaire survey effectively and comprehensively reflect the level of health behaviours in clinical nurses, which provides a scientific reference for determining the intervention target.

## Background

Nurses play a central role in the health care system and are one of the main labour forces. Nurses, as health care providers, protectors, disseminators, coordinators, decision-makers and teachers, provide different health services in different environments [1]. Nurses in China account for nearly one-fifth of the world’s nurses [2, 3]. Nurses are the first to respond to different health-related conditions and can promote health recovery and prevent diseases [1]. More importantly, nurses’ own health behaviours can greatly influence the effectiveness of the health interventions delivered to their patients [4]. Since nurses play an indispensable role in the hospital, it is particularly important to ensure that they do not quit their jobs due to physical problems.

Health promotion behaviours refer to all behaviours that guide individuals, families, communities, and societies to promote peace, happiness, and the realization of their health potential, including physical behaviours, such as diet, nutrition, exercise, and health responsibility, as well as psychological behaviours, such as spiritual growth, stress management, and interpersonal relationships [5, 6]. The content of health promotion behaviours is guided by health promotion, which is an indicator of individuals’ efforts to achieve a healthier state [7]. The World Health Organization (WHO) points out that there is a close correlation between health and lifestyle and that health promotion is about empowering individuals and populations to make healthier choices and follow lifestyles that promote physical and mental health [8].

In particular, nurses engaged in clinical nursing work often do not have a healthy lifestyle due to the special nature of their work [9], so more health promotion programs are needed to improve their poor lifestyle habits. Relevant studies [10, 11] have shown that diet, physical activity or stress interventions for clinical nurses can improve their well-being, their health status and the quality of their nursing work.

Heavy workloads, complex interpersonal relationships, negative stimulation due to the pain and death of patients, stress caused by worrying about errors and accidents [12] and physical and mental fatigue caused by frequent shift work [13] are all risk factors affecting the physical and mental health of nurses. In terms of physical health, nurses have an increased incidence of insomnia, obesity, stomach diseases, endocrine disorders, varicose veins and even breast cancer due to these risk factors [14–18]. In terms of mental health, nurses experience anxiety and depression due to changes in hospital units or departments, heavy workloads and long-term work in stressful and uncertain environments [19]. This not only reduces clinical nurses’ work efficiency but also leads to job errors and the deterioration of interpersonal relationships, eventually leading to health problems and job burnout [20]. In addition, studies have shown that high job burnout and low health levels also increase the separation rate of nurses [21], which has an impact on hospital clinical nursing work. Compared with other populations [22], clinical nurses may have an increased number of poor lifestyle habits [13–23], such as an unreasonable diet and reduced physical activity levels, which makes them prone to various health problems [24].

At present, the health promotion behaviours of clinical nurses urgently need to be widely considered, and interventions are needed to improve the health level and reduce the incidence of diseases among clinical nurses [25–27]. Screening and evaluation is the most important first step before intervention, so an appropriate evaluation tool is necessary. However, there are few scales to measure the health behaviours of clinical nurses working in hospitals in China. Initially, Walker and others [28] developed Health Promoting Lifestyle Profiles (HPLP) to assess people’s health-promoting lifestyles. Subsequently, Pender et al. developed the Health Promoting Lifestyle Profile II (HPLP-II)[29], which is mainly used to assess whether individuals have a healthy lifestyle in the general population. Later, Sun, Huang and Ling developed an improved Chinese version of the HPLP [30]. Although the three scales differ in the number of items, what they measure is relatively similar. In contrast, the existing health behaviour scales are mostly developed by Western countries and focus on Western cultural habits and lifestyles, and these scales are universal scales, lacking reference for occupational specificity and cultural differences. Recently, Woynarowska-Sołdan et al. developed a validated instrument called the Positive Health Behaviours Scale [31], which evaluates the health promotion behaviours of clinical nurses from four aspects: nutrition, physical activity, relaxation and behaviours related to mental health, and preventive behaviours. Each dimension of the scale comprehensively presents different aspects of health promotion behaviours. According to the background of low self-care consciousness and high prevalence rate of clinical nurses, the scale fully considered the preventive behavior and lifestyle of clinical nurses, and the nurses’ health behaviours scale was reasonably constructed. At present, there is no study reporting on the reliability and validity of the translated version of this scale. The results of the evaluation of this scale will be helpful for clinical nursing managers to develop interventions to improve the health behaviours of clinical nurses and compare differences before and after interventions.

The aim of this study was to translate the PHBS into Chinese and further cross-culturally adaptation and to validate its psychometric properties in clinical nurses.

## Methods

### Study design and participants

A cross-sectional survey was adopted to evaluate the Chinese version of the PHBS. A total of 633 nurses from 3 Grade A hospitals in Liaoning Province were selected by convenience sampling from September 2021 to March 2022. The inclusion criteria were registered nurses with at least 1 year of clinical work experience who provided informed consent and volunteered to participate in the study. The exclusion criteria were as follows: practice, study, and rotation nurses (practice nurses refer to nursing graduates who work in hospitals for 9 months before taking the national nurse practice exam and do not have the right to work independently during this period; study nurses are students who go to the hospital to study during the school year due to course needs; and rotating nurses are nurses who rotate throughout the wards). The respondents were interviewed face-to-face in the department by the investigator. According to the rough estimation method to determine sample size, the sample size required for scale reliability and validity tests must be 5 ~ 10 times [32] the number of scale items. To ensure the stability of the factor structure, CFA should include at least 300 participants [33], and a larger sample size should be considered. In this study, there were 29 items in the Chinese version of the PHBS. The reference sample size should be 10 times the number of items in the scale, but considering that the sample loss rate may be 20%, it was estimated that 348 nurses should be included in this study. A total of 633 clinical nurses were recruited for this study.

### Instruments

#### General demographic characteristics questionnaire

According to the purpose of the study, the researchers designed a general demographic characteristics questionnaire, including age, educational level, marital status, number of working years, position titles, personnel relations and self-assessed health.

**Table 1:** Frequency distribution of demographic characteristics (n = 633)

Factors	Group	n	%
Age	20~	435	68.7
	30~	169	26.7
	40~	29	4.6
Educational level	Junior college education	59	9.3
	Undergraduate education	511	80.7
	Postgraduate education or above	63	10.0
Marital status	Unmarried	444	70.1
	Married	189	29.9
Working years	1~	488	77.1
	10~	127	20.1
	20~	18	2.8
Positional titles	Primary nurse	372	58.8
	Nurse practitioner	166	26.2
	Nurse-in-charge or above	95	15.0
Personnel relations	Contract nurses*	368	58.1
	Formal nurses*	209	33.0
	Other	56	8.8
Health self-assessment status	Particularly good	360	56.9
	Good	238	37.6
	Poor	29	4.6
	Particularly poor	6	0.9

Note: *Contract nurses are hospitals and nurses sign labor contracts. Formal nurses are recruited by the local health bureau, and the work is stable.

#### Positive health behaviors scale (PHBS)

The Positive Health Behaviours Scale for clinical nurses developed by Woynarowska-Sołdan et al. [31] consists of 29 items covering four dimensions: nutrition (nine items), physical activity (four items), relaxation and behaviours related to mental health (seven items), and preventive behaviours (nine items). Participants’ behaviour is scored on a four-point scale ranging from 0 for “never or almost never” to 3 for “always or almost always”. The PHBS total score ranges from 0 to 87, and the higher the score is, the higher the level of healthy behaviours. Cronbach’s α coefficient of the original scale was 0.844, while that for each dimension ranged from 0.623 to 0.761. In the original scale, four common factors were forcibly extracted to explain 38% of the total variance, with GFI = 0.87 and RMSEA = 0.07.

**Table 2:** Mean (SD) scores with skewness and kurtosis, item analysis for Chinese version of the positive health behaviours scale

Item	Item score (SD)	Critical ratio	Item-total correlation	Cronbach’s Alpha if item deleted	Skewness	Kurtosis
1	2.1(0.7)	17.577	0.614	0.925	-0.175	-0.781
2	2.1(0.7)	18.042	0.611	0.925	-0.351	-0.610
3	2.1(0.8)	19.957	0.621	0.925	-0.152	-1.105
4	2.2(0.7)	18.228	0.612	0.925	-0.310	-0.645
5	1.9(0.8)	19.956	0.614	0.925	-0.018	-1.004
6	1.9(0.7)	19.636	0.649	0.925	0.011	-0.906
7	2.0(0.7)	19.133	0.636	0.925	-0.102	-0.777
8	2.0(0.7)	18.168	0.638	0.925	-0.122	-0.689
9	2.0(0.8)	16.099	0.577	0.926	-0.099	-1.036
10	1.6(0.8)	10.628	0.463	0.928	0.120	-0.605
11	1.6(0.9)	12.108	0.489	0.927	0.038	-0.679
12	1.7(0.8)	12.084	0.489	0.927	-0.005	-0.566
13	1.6(0.9)	12.004	0.460	0.928	0.002	-0.708
14	2.1(0.7)	13.746	0.510	0.927	-0.164	-0.821
15	1.9(0.7)	16.397	0.590	0.926	-0.052	-0.622
16	2.0(0.7)	14.906	0.534	0.926	-0.147	-0.687
17	2.0(0.7)	15.217	0.552	0.926	0.022	-0.902
18	2.0(0.7)	15.197	0.565	0.926	-0.003	-0.634
19	2.0(0.7)	15.317	0.550	0.926	-0.003	-0.907
20	2.0(0.8)	13.940	0.521	0.927	-0.108	-0.884
21	1.9(0.8)	14.195	0.536	0.926	-0.416	-0.326
22	2.1(0.7)	17.414	0.610	0.925	-0.209	-0.762
23	2.3(0.7)	17.691	0.590	0.926	-0.426	-0.856
24	2.0(0.8)	20.880	0.636	0.925	-0.295	-0.826
25	2.0(0.7)	18.242	0.645	0.925	-0.302	-0.442
26	2.1(0.7)	19.144	0.632	0.925	-0.326	-0.571
27	2.0(0.8)	19.749	0.639	0.925	-0.183	-0.881
28	2.0(0.8)	18.800	0.643	0.925	-0.244	-0.543
29	2.2(0.7)	16.698	0.578	0.926	-0.316	-0.837

### Procedures

#### Scale translation and cross-cultural adaptation procedure

With the permission of Professor Woynarowska-Sołdan [31], we translated and cross-culturally adjusted the scale. The PHBS was translated into Chinese by the Brislin method [34]. The specific steps are as follows: (1) Translation: the researcher and a nursing graduate student translated the original scale to form a translated version; (2) Correction: another researcher retranslated the Chinese version into English, and two nursing experts compared the translated version, discussed and evaluated the translation quality, and revised the professional terms to form the first draft; and (3) Back translation: to achieve semantic equivalence, a nonmedical researcher was invited to back translate the first draft into English and form the final Chinese version of the PHBS. Subsequently, 30 community clinical nurses were randomly selected to evaluate the clarity and agreement of the Chinese version of the PHBS.

**Fig. 1:**
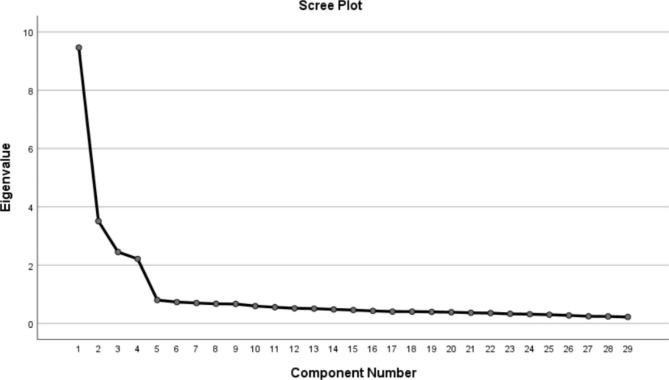
Screen plot of exploratory factor analysis for Chinese version of the Positive Health Behaviours Scale

#### Data collection procedure

Before distributing the questionnaires on site, the researcher first obtained the consent of the manager of the nursing department and the head nurses of related departments and avoided the department’s busy work hours. With the assistance of the nursing department manager, the researcher and two other trained investigators went to 3 Grade A hospitals in Liaoning Province. A convenience sampling method was used to distribute questionnaires to nurses in the departments who met the inclusion and exclusion criteria and to inform the nurses of the purpose and significance of the study and matters that should be paid attention to when filling out the questionnaires. After completing the questionnaire, the data were collected. A total of 640 nurses completed the questionnaire. Abnormal questionnaires with obvious regularity or confusing logic were eliminated from the data (for example, the answers had the same choices or the answers were contradictory). Ultimately, 633 valid questionnaires were collected, with an effective recovery rate of 98.9%. Two weeks later, 60 nurses were randomly selected for a second survey to assess the retest reliability of the scale.

### Data analysis

SPSS 26.0 and Amos 22.0 were used for statistical analysis. The measurement data are represented by mean values (standard deviation, SD), and the enumeration data are described by percentages. Data were considered normally distributed when the skewness and kurtosis values of the items were between − 2 and + 2 [35]. Item analysis, validity analysis, and reliability analysis of the Chinese version of the PHBS were performed in our study.

**Table 3:** Factor loadings of exploratory factor analysis for the Chinese version of the Positive Health Behaviours Scale

Item	Factor 1	Factor 2	Factor 3	Factor 4
21. In autumn and winter, I will supplement vitamin D	0.593	-	-	-
22. I will avoid excessive sun exposure	0.687	-	-	-
23. I brush my teeth at least twice a day	0.776	-	-	-
24. I check my teeth every six months	0.795	-	-	-
25. I measure my blood pressure every three months	0.775	-	-	-
26. I will be vaccinated against influenza to prevent disease	0.739	-	-	-
27. I have a breast self-examination once a month	0.825	-	-	-
28. I have a smear test (female) or blood test for prostate specific antigen (male) at least every 3 years or less	0.784	-	-	-
29. When I am ill, I will follow the doctor’s advice and receive treatment	0.733	-	-	-
1. I have regular meals every day	-	0.713	-	-
2. I have the habit of eating breakfast	-	0.719	-	-
3. I eat fruit every day	-	0.771	-	-
4. I eat vegetables every day	-	0.718	-	-
5. I drink at least two glasses of milk or yogurt every day	-	0.798	-	-
6. I limit the intake of animal fat every day	-	0.762	-	-
7. I limit my intake of salt every day	-	0.729	-	-
8. I limit my sugar intake every day	-	0.751	-	-
9. I don’t eat snacks between meals	-	0.696	-	-
14. I sleep at least 6–7 hours every night	-	-	0.710	-
15. I work and rest regularly every day	-	-	0.723	-
16. I spend at least 20 minutes a day relaxing	-	-	0.735	-
17. I can cope with pressure well	-	-	0722	-
18. Facing myself and the outside world, I can always maintain a positive attitude	-	-	0.650	-
19. When I encounter difficulties, I will ask others for help	-	-	0.733	-
20. I get together with my friends or colleagues once a month	-	-	0.639	-
10. I do at least 30 minutes of moderate or vigorous exercise every day	-	-	-	0.837
11. I do strength exercises of major muscle groups at least twice a week (e.g. sweeping the floor, carrying heavy bags, climbing stairs, exercising abdominal muscles)	-	-	-	0.835
12. In daily life, I will take the initiative to increase physical activity or physical labor (for example, walking instead of taking a car, climbing stairs instead of taking an elevator)	-	-	-	0.775
13. I limit the time I watch TV every day	-	-	-	0.766

### Items analysis

The total scores of the Chinese version of the PHBS were ranked from high to low and divided into low and high subgroups by 27% and 73% quantile boundaries. The independent-samples T test was used to compare the differences in each item between the high-score group and the low-score group. A critical ratio > 3.000 [36] indicated that the discriminability of the item was high. Whether each item of the Chinese version of the PHBS could be retained was assessed by analysing the item-total correlation and Cronbach’s alpha coefficient of the deleted item. The item-total correlation was judged with 0.4 as the inclusion criterion [37].

**Fig. 2:**
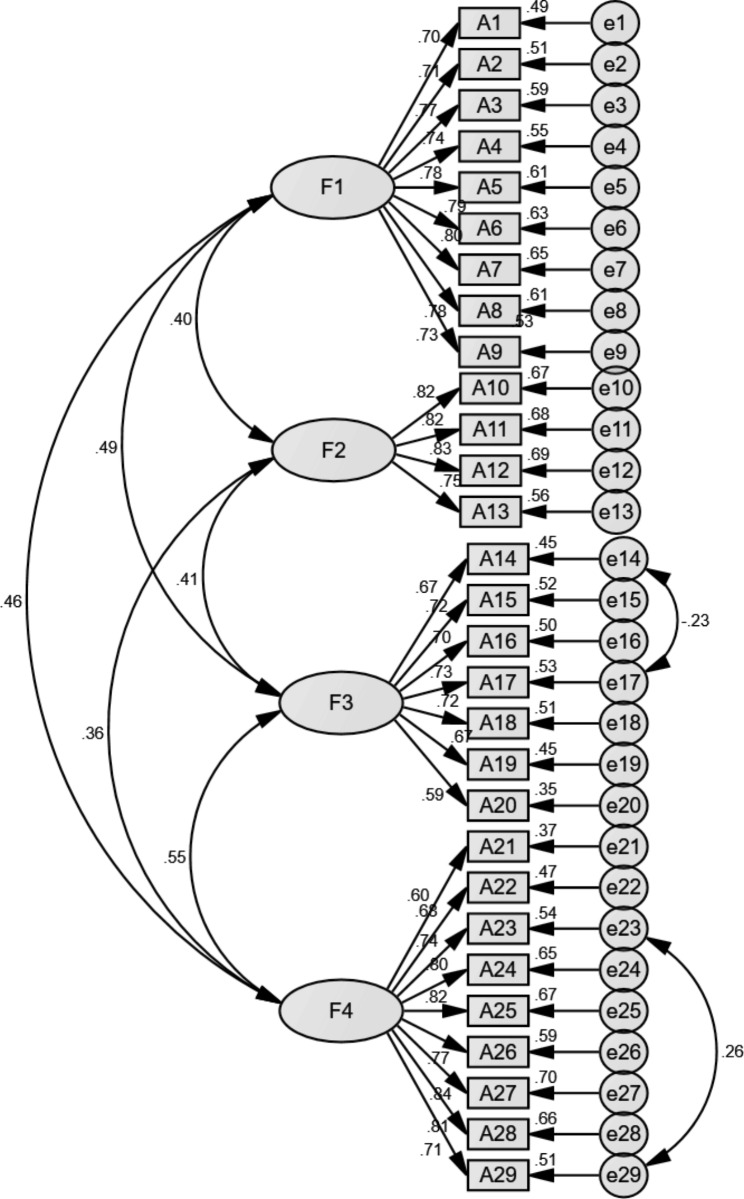
Standardized four-factor structural model of the Chinese version of the Positive Health Behaviours Scale (n = 317)

### Validity analysis

Seven nursing specialists were invited to access each item in the translated scale from the perspective of content importance. The Delphi method was used to calculate the item content validity index (I-CVI) and scale content validity index (S-CVI). A 4-point Likert scale was used to evaluate the content correlation of each item of the Chinese version of the PHBS, ranging from 1 = irrelevant to 4 = highly relevant. The I-CVI refers to the proportion of the number of experts who gave a score of 3 or 4 points for an item to the total number of experts; the S-CVI is the mean I-CVI of all items in the scale. An I-CVI > 0.780 and an S-CVI > 0.900 indicated better content validity [38].

The total sample was randomly divided into sample 1 and sample 2 by a simple random method. Exploratory factor analysis (EFA) and confirmatory factor analysis (CFA) were performed to evaluate the structural validity. Factor analysis was performed on the data, and the applicable conditions were as follows: the calculated value of the Bartlett sphericity test was significant (P < 0.05) and the KMO value was > 0.60 [36]. The principal component analysis method was used to extract common factors with eigenvalues > 1 by rotating through the varimax method and deleting items with factor loads < 0.50 [36]. CFA was implemented to confirm the hypothesized factor model, and the maximum likelihood method was used for estimation.

**Table 4:** Fit indices of the Chinese version of the Positive Health Behaviours Scale model

Fit indices	χ^2^/df	RMSEA	RMR	GFI	NFI	TLI	CFI	IFI
Model modification	1.363	0.034	0.023	0.902	0.909	0.971	0.974	0.974
range [43]	< 3.000	< 0.080	< 0.050	> 0.900	> 0.900	> 0.900	> 0.900	> 0.900

**Note**: RMSEA = the root mean square error of approximation, RMR = root mean residual, GFI = goodness-of-fit index, NFI = the normed fit index,TLI = the tucker lewis index, CFI = the comparative fit index, IFI = the incremental fit index

### Reliability analysis

Cronbach’s α coefficients, Guttman split-half reliability and test-retest reliability were analysed to validate the reliability of the translated scale. Cronbach’s α coefficients of the total scale and each dimension of the scale were calculated, and a value of 0.70 was taken as the acceptable standard for the reliability coefficient [39]. Split-half reliability was assessed by dividing the scale items into two halves in parity order and calculating the correlation between the scores of the two parts. Two weeks later, 60 clinical nurses were retested using the translated scale and the correlation with the results of the first measurement was analysed to evaluate the stability and consistency throughout the data collection period. Since the Pearson correlation coefficient is often higher than the true reliability, the intraclass correlation coefficient (ICC) was also used to calculate the consistency of the two measurements.

## Results

### Scale translation and cross-cultural adaptation

According to experts’ opinions, the Chinese version of the PHBS was revised and improved. The details were as follows: Item 5, “I drink at least 2 glasses of milk, kefir or yogurt daily” was changed to “I drink at least two glasses of milk or yogurt every day”. In China, it is widely believed that kefir is a type of yogurt, so it was deleted. Item 28, “I have a smear test at least once every 3 years or more often as prescribed by a physician” was changed to “I have a smear (females) or PSA blood test (males) at least every 3 years or less”. In the original scale, only female nurses were included; in China, the number of male nurses accounted for a certain proportion of nurses working in the hospital. We made certain modifications to this item to make it applicable to male nurses.

**Table 5:** Reliability analysis for the Chinese version of the Positive Health Behaviours Scale

The scale and its dimension	Cronbach’s Alpha	Split-half reliability	Test-retest reliability
The Positive Health Behaviors Scale	0.928	0.953	0.891
Nutrition	0.920		
Physical activity	0.864		
Relaxation and behaviors related to mental health	0.860		
Preventive behaviours	0.919		

### Descriptive Statistics

A total of 640 questionnaires were collected in this study, and 633 valid questionnaires were ultimately obtained after the elimination of 7 invalid questionnaires. A total of 68.7% of the participants were aged 20 to 29 years. The number of participants with an undergraduate education accounted for 80.7% of the sample. Participants who were unmarried accounted for 70.1% of the sample; 77.1% of the participants had been engaged in clinical nursing work for 1 to 9 years. There were more participants (58.8%) who obtained the professional title of primary nurse. A total of 58.1% of the participants were contract nurses; 56.9% of the participants thought that their self-assessed health status was particularly good. Table 1 lists all the characteristics of the participants.

### Item Analysis

The critical ratio of 29 items in the translated scale ranged from 10.628 to 20.880, and the differences in each item in the high-score and low-score groups were statistically significant (P < 0.001). The correlation coefficient between the score of each item and the total score of the translated scale was 0.460~0.649. Cronbach’s α coefficient of the translated scale was 0.928, and after deleting any item, Cronbach’s α coefficient of the translated scale ranged from 0.925 to 0.928, without any specific value. The mean (SD) item score and skewness and kurtosis values of the Chinese version of the PHBS are shown in Table 2. The skewness and kurtosis values showed that the detected dataset conformed to a normal distribution.

### Validity analysis

#### Content Validity Analysis

Seven experts were invited to rate the importance of each item on the scale. The results showed that the content validity index at the scale level was 0.956, and the content validity index at the item level was 0.857 ~ 1.000.

#### Exploratory factor analysis

The KMO value was 0.928, χ^2^ = 4905.714, and P < 0.001 using the Bartlett sphericity test, which indicated that the partial correlation between items was weak, and EFA could be performed in this study. Four common factors with eigenvalues > 1 were extracted by principal component analysis and orthogonal rotation of factors by the varimax method. The explained variances were 18.80%, 18.70%, 13.54% and 9.83%, respectively, explaining 60.81% of the total variance in the variables. The loading of each item on its factor was > 0.50, so no item was deleted. The factor loading for each item is shown in Table 3. Four principal component factors were selected according to the descending slope of the eigenvalues in the scree plot. Figure 1 shows the scree plot.

### Confirmatory factor analysis

Figure 2 shows the results of CFA. In Amos, the maximum likelihood method was used to conduct CFA on another part of the data of the scale (n = 317), and the initial model was revised according to the modification indices (MIs) as follows: e14 and e17, e23 and e29, respectively. The fitness index model modification is shown in Table 4. The results of each fitted indicator after correction showed that the χ^2^/df was 1.363, the GFI was 0.902, the NFI was 0.909, the TLI was 0.971, the CFI was 0.974, and the IFI was 0.974. The RMSEA was 0.034, and the RMR was 0.023. Each fitted indicator of CFA was within the reference range.

### Reliability analysis

Cronbach’s α coefficient of the translated scale was 0.928, and the values of the four dimensions ranged from 0.860 to 0.920. The split-half reliability value was 0.953. Sixty clinical nurses were randomly selected for a retest 2 weeks later; the retest reliability value was 0.891 (Table 5) and the ICC was 0.885.

## Discussion

According to relevant studies [40], due to the existence of multidimensional stress, nurses may adopt ineffective coping mechanisms (such as overeating, reducing physical activity, etc.) to deal with work-related stressors, and these unhealthy behaviours seriously affect their physical and mental health, thus leading to the occurrence of diseases. Healthy lifestyles among nurses are receiving increasing attention from managers. In China, scales used to measure nurses’ health behaviours are all universal, and a large number of items may increase nurses’ workloads. Therefore, accurate and appropriate tools that can be applied to evaluate healthy lifestyle behaviours in clinical nurses are necessary. The Positive Health Behaviours Scale (PHBS) was developed by Woynarowska-Soredan in 2018. It is a research tool developed to evaluate healthy lifestyles among clinical nurses, and it comprehensively evaluates positive health behaviours from four aspects: nutrition, physical activity, preventive behaviours, and relaxation and behaviours related to mental health. The content of preventive behaviour was added to this scale, which can more accurately measure the health status of clinical nurses under the continuous development of the current era and meet the requirements of China’s advocacy for a prevention-oriented healthy lifestyle. The Chinese version of the PHBS strictly followed the Brislin principle [34] in the translation process and literal translation, back translation and cultural adjustment procedures were carried out. After statistical analysis, the results showed that the Chinese version of the PHBS has good reliability and validity and can be used to evaluate nurses’ health behaviours and improve their health awareness. It provides a reliable assessment tool for further in-depth and comprehensive understanding of nurses’ health promotion behaviours and their impact on nurses and can help guide clinical nursing managers to develop effective intervention measures.

### Item analysis

The critical ratios of the Chinese version of the PHBS were all within the standard range [36], indicating that each item of the scale had the ability to identify the health behaviour level of different survey subjects. The results of the correlation coefficient method showed that each item had a high correlation with the dimension [37]. After deleting items, the Cronbach’s α coefficient of the translation scale did not increase, indicating a strong correlation between items and high internal consistency. This means that all 29 items in the Chinese version of the PHBS can be retained with good discrimination.

### Validity analysis

Content validity refers to the extent to which a concept measured by a researcher is reflected by questionnaire items [41]. In this study, the I-CVI and S-CVI were within the reference value range [38], indicating that this scale has good content validity. Therefore, the results showed that the items of the scale could better reflect the measured content. Structural validity refers to whether the multi-index measurement of an objective thing has a professional ideal structure [42]. When the factor load value of each item to the corresponding common factor is appropriate and the cumulative explanatory variation is > 40%, the structure validity can be considered to be good. The orthogonal rotation method of maximum variance was used in this study, and a factor load ≥ 0.50 was the test standard. In this study, 4 common factors were extracted from the EFA without deleting any item, and the items of each dimension were in accordance with the original scale [31]. The EFA results divided 29 items in the translated scale into four factors, and the cumulative variance contribution rate was 60.81%, which is higher than that of the original scale (38%), indicating that the extracted common factors had good interpretability for the dimensions. CFA is a measurement of whether the relationship between a factor and its corresponding index conforms to the research’s design theory. The CFA results in this study showed that χ^2^/df ≤ 3, RMSEA < 0.08, RMR < 0.05 and other relative fitting indices > 0.90, and the fitting value reached the ideal fitting standard [43]. Further CFA results indicated that the scale structure was scientific and stable and had good structural validity.

### Reliability analysis

Reliability refers to the reliability of the measured data [44], and common indicators include internal consistency and test-retest reliability. It is generally considered that a Cronbach’s α coefficient above 0.7 is acceptable, and 0.8~0.9 indicates good reliability [39, 41]. In this study, Cronbach’s α coefficient of the Chinese version of the PHBS was 0.928, which was higher than the results of the Polish version [31], indicating that the scale has good internal consistency and high credibility. Moreover, the test-retest reliability was also good, which proved the cross-time stability of the Chinese version of the PHBS. Consequently, the Chinese version of the PHBS has good reliability.

### Limitations

There are some drawbacks in this study that need to be considered. First, only part of the nursing population completed the scale, which may affect the representativeness and universality of the survey results. Second, due to the heavy workload of nurses, this study failed to measure the predictive validity of the scale and could not evaluate the impact of nurses’ health behaviours. Third, convenience sampling was used in this study, which may make the determination of sample units unrepresentative. Finally, this study relied on principal component factor analysis, which has a certain ambiguity in its interpretation, which may lead to overestimation of the number of common factors.

## Conclusions

In conclusion, the Chinese version of the PHBS formed in this study has a clear description, a short completion time, and moderate reliability and validity in hospitals. This method can effectively evaluate the level of positive health behaviours in clinical nurses and has strong operability. It can be applied to clinical scientific research. Clinical nursing managers can understand the health behaviours of nurses according to the measurement results of the scale and take corresponding measures to improve nurses’ awareness of disease prevention and health care to improve the quality of nursing work.

## Data Availability

The datasets used and/or analysed during the current study are available from the corresponding author on reasonable request.
